# Development and Validation of the Life for Low Vision Questionnaire (LIFE4LVQ) Using Rasch Analysis: A Questionnaire Evaluating Ability and Independence

**DOI:** 10.3390/jcm12072549

**Published:** 2023-03-28

**Authors:** Stavroula Almpanidou, Diamantis Almaliotis, Leonidas Karamitopoulos, Fotios Topouzis, Anastasios-Georgios Konstas, Georgios Labiris, Theodoros Dardavesis, Konstantinos N. Fountoulakis, Konstantinos Ch. Chatzisavvas, Vasileios Karampatakis

**Affiliations:** 1 Laboratory of Experimental Ophthalmology, School of Medicine, Aristotle University of Thessaloniki, 54124 Thessaloniki, Greece; 2 1st Department of Ophthalmology, Aristotle University of Thessaloniki, 54124 Thessaloniki, Greece; 3 Department of Ophthalmology, University Hospital of Alexandroupolis, 68100 Alexandroupolis, Greece; 4 Department of Hygiene, Social-Preventative Medicine and Medical Statistics, School of Medicine, Aristotle University of Thessaloniki, 54124 Thessaloniki, Greece; 5 Third Department of Psychiatry, Faculty of Medicine, School of Health Sciences, Aristotle University of Thessaloniki, 54124 Thessaloniki, Greece; 6 mSensis S.A., VEPE Technopolis, Bld C2, 55535 Thessaloniki, Greece; 7 Department of Electrical and Computer Engineering, University of Western Macedonia, ZEP Campus Kozani, 50100 Kozani, Greece

**Keywords:** low vision, functionality, ability, independence, Rasch analysis, PROMs

## Abstract

Low vision (LV) has a substantial impact on an individual’s daily functionality and patient-reported outcome measures (PROMs) are increasingly incorporated into the evaluation of this problem. The objective of this study was to describe the design of the new “Life for Low Vision Questionnaire (LIFE4LVQ)”, as a measure of daily functionality in LV and to explore its psychometric properties. A total of 294 participants completed the LIFE4LVQ and the data were subjected to Rasch analysis to determine the psychometric properties of the questionnaire, including response category ordering, item fit statistics, principal component analysis, precision, differential item functioning, and targeting. Test–retest reliability was evaluated with an interval of three weeks and intraclass correlation coefficients (ICC) were used. The correlation between the questionnaire score and Best Corrected Visual Acuity (BCVA) was examined using Spearman’s correlation coefficient. Rasch analysis revealed that for most items the infit and outfit mean square fit values were close to 1, both for the whole scale and its subscales (ability and independence). The separation index for person measures was 5.18 with a reliability of 0.96, indicating good discriminant ability and adequate model fit. Five response categories were found for all items. The ICC was 0.96 (p < 0.001; 95% CI, 0.93–0.98), suggesting excellent repeatability of the measure. Poorer BCVA was significantly associated with worse scores (rho = 0.559, p < 0.001), indicating excellent convergent validity. The functional, 40-item LIFE4LVQ proved to be a reliable and valid tool that effectively measures the impact of LV on ability and independence.

## 1. Introduction

Vision is a vital sense responsible for an individual’s daily functionality, having at the same time an impact on physical and emotional well-being [1]. According to the World Health Organization (WHO), Low Vision (LV) is defined as a Best Corrected Visual Acuity (BCVA) of less than 20/70 or a visual field of equal to or less than 20° in the better eye after refractive correction and medical or surgical treatment, if necessary [2]. It is estimated that 285 million people live with Visual Impairment (VI) worldwide, of which 246 million are reported as having LV [3]. Moreover, the prevalence of LV is anticipated to increase significantly over the next decades due to the increasing age of the population worldwide [4].

LV constitutes a significant public health burden [5,6]. Although the clinical examination is valuable, it may not incorporate real-world difficulties that LV patients face and does not capture the burden of VI from the patient’s viewpoint. Specifically, the clinical examination has been found to have a relatively weak correlation with Patient-Reported Outcome Measures (PROMs); visual function (which describes the eyes’ function) is different from functional vision (which describes a person’s function), and also from vision-related Quality of Life—QoL (individual satisfaction) [7,8,9,10,11]. This has led to the development of questionnaires that evaluate perceived QoL, as well as functionality in daily life. However, “Functional Vision” is very often confused with the “vision-specific QoL” [12] due to some kind of overlap between these two concepts [13]. Specifically, functional vision describes the residual ability of a person to perform visually dependent activities [9,10], while the term “Vision-related QoL” also includes emotional well-being, social relationships, and concerns, as they are affected by vision [14]. According to Collenbrader, questions relating to functional vision and QoL should be evaluated separately [8].

To date, most vision-related questionnaires have been developed to measure constructs of QoL rather than functionality, and the target population is mainly individuals with “mild to moderate visual impairment” instead of the “low vision” population [15,16,17,18,19]. Using outcome measures that confound constructs such as well-being or QoL with functionality may interfere with the accurate evaluation of the effectiveness of rehabilitation strategies or other interventions [20]. Regarding questionnaires that assess functionality in daily life, interestingly, the majority of them target only specific conditions such as cataract, a treatable cause of vision loss [16], excluding other causes of irreversible vision loss [15,16,17,18,19]. Furthermore, most of these already existing questionnaires were developed based on classic test theory and are now being re-validated by the implementation of modern statistical techniques (Rasch analysis) [16,21]. Moreover, the existing functional questionnaires include items asking individuals about their ability to read from paper sheets; however, reading habits have changed over time with the advent of digital media, meaning that these questionnaires do not cover the use of digital devices. Wu et al. found that digital reading is time-demanding for adults with LV, which indicates their difficulty in using new technologies [22].

Within this context, there is a need to develop a new questionnaire assessing the daily life functionality in individuals who belong to the LV spectrum, updated with new items related to technology and using modern and validated statistical methods from conception. Moreover, the vision-related functional questionnaires measure only the ability to perform/ease of performing a task, as the only parameter to assess performance in activities of daily living [16,17,18]. According to Massof and colleagues, there are two discriminated attributes (construct variables) of performing an activity of daily living in the LV population: the “functional ability’’ and the value of ‘’independence’’, through assessing the importance of a specific task in a person’s life [23].

According to this conceptual framework, the objective of the current study was to develop and explore the psychometric properties of the new Life for Low Vision Questionnaire (LIFE4LVQ) targeting the evaluation of vision-related functionality in LV patients, using both ability and independence as variables of the measurement. The LIFE4LV questionnaire is one of the first questionnaires to employ Rasch analysis from conception. Rasch analysis is a probabilistic model based on modern test theory which compares the difficulty of items (item difficulty) with the relative abilities of the respondents (person ability) [24,25,26,27]. Based on the research hypothesis of the current study, we assumed that the new questionnaire would capture two main variables of functionality in LV: ability and independence.

## 2. Materials and Methods

### 2.1. Participants

A total of 534 participants were referred to our outpatient unit at the Laboratory of Experimental Ophthalmology, at the Aristotle University of Thessaloniki, running the LIFE4LV program for patients with VI (NCT05184036). All participants were referred with their diagnosis. The inclusion criteria were as follows: participants had to be Greek, literate, with a VI diagnosis due to untreatable causes, ≥18 years of age, and without significant hearing or cognitive impairment (as measured using the Minimental test (MMSE44)) [28]. Patients with severe systemic comorbidities were excluded from the study. Furthermore, patients with treatable causes of VI, such as cataract and or uncorrected refractive errors, were also excluded from the study.

After screening for eligibility criteria, 454 participants were eligible to participate. Specifically, 110 participants were recruited in the content development phase, during focus groups; another 50 participants were administered the pre-final version of the questionnaire. The psychometric properties of the questionnaire were further evaluated in 294 participants who were divided into 3 groups: 20 participants with mild VI (group 1), 252 patients with LV according to the WHO criteria [4] (group 2), and 22 legally blind individuals (group 3). All those invited for the interview (after screening for eligibility criteria) answered the questionnaire (response rate 100%).

All participants underwent a visual acuity examination by an experienced examiner using the Early Treatment Diabetic Retinopathy Study (ETDRS) chart (Precision Vision, La Salle, IL, USA). A detailed medical history, demographic characteristics, and educational level were also recorded. Written informed consent was obtained by all participants in the study after an explanation of the nature of the study. The study was approved by the Committee for Bioethics and Ethics, Medical Department, Aristotle University of Thessaloniki (code#1.60/21.11.2018) and adhered to the tenets of the Declaration of Helsinki.

### 2.2. Design of the LIFE4LV Questionnaire

Phase A. Content Development through a literature review and focus groups

Following the recommendations of the FDA suggesting that a conceptual framework helps guide the development of a PROM [29], the new questionnaire was designed to investigate two constructs: (a) the ability and (b) the independence of the LV population. The general principles of developing a measurement were utilized [12,30]. The previously published literature in the field was exploited [31,32,33] in addition to meetings with experts in the field, to select vision-related items from existing instruments, such as (1) the National Eye Institute Visual Function Questionnaire-25 [34,35], (2) Low Vision Quality of Life Questionnaire (LVQOL) [36], (3) Macular Disease on Quality of Life (the MacDQoL) [37], (4) Visual Function-14 [38,39], (5) Activities of Daily Vision Scale [40,41], (6) Visual Activities Questionnaire [24], (7) Low Luminance Questionnaire [42], and (8) Impact of Visual Impairment [43,44,45].

The item identification was based on the approach proposed by Massof and colleagues [23]. Focus groups of patients with LV (11 focus groups including 10 LV patients each time) were organized. The participants were assessed and interviewed by a group of experts (five ophthalmologists, one epidemiologist, one psychiatrist, etc.). We asked the participants to report up to three daily activities that were affected by their visual impairment and that were significant to them (Table 1). Furthermore, we investigated the patients’ feedback regarding the items that were selected based on the literature, and whether they were relevant or not. Activities that had not been engaged in within the past 6 months for reasons unrelated to vision were not evaluated. This process enabled the identification of specific activities that were more prone to be affected in LV patients and were selected to match the particular needs of LV patients.

The initial version of the questionnaire consisted of 58 items. Listening to music was removed from the list of possible activities for item construction since it is not a vision-related activity. Furthermore, sex life, pet care, gardening, needlecraft, volunteering, spirituality, and attending the theater were also removed because they were found not to apply to most respondents and probably to a large proportion of the population (Table 1.)

Phase B. Scale Refinement

Using information from the previous phase and interviews, we developed a 49-item questionnaire. Several modifications were made using the quality assessment criteria proposed by Pesudovs and colleagues to improve the item set, the scoring method, and the whole instrument [12]. The items were phrased in simple language and kept as clear and brief as possible [12,46]. According to Streiner and Norman, the items should be phrased at the comprehension level of a twelve-year-old person [46]. Cognitive debriefing interviews were also conducted with a sample of 10 LV participants [47]. Participants were asked to describe in their own words what each item was asking and to report any problems with the way the questions were expressed by comparing several ways of expressing of the same content. The process was repeated until no further issues emerged. The majority of these participants reported that the LIFE4LVQ was in general comprehensible and relevant to their vision-related daily living.

A pilot study evaluating the properties of a first draft of the LIFE4LVQ was also conducted with 50 participants: 15 with mild VI, 10 legally blind, and 25 with LV, who were randomly picked from these discriminant visual impairment groups. The questionnaire was paper-based and was administered through face-to-face interviews. The preliminary findings indicated that the draft form had good internal consistency (Cronbach’s alpha > 0.8) and that the questionnaire was sensitive to subgroup differences by measuring vision-related ability and independence.

Phase C. Psychometric analysis in the LV group

The 49-item version of the LIFE4LV questionnaire (Supplementary Table S1) was administered to 252 LV patients for further evaluation of its psychometric properties. Furthermore, 20 individuals with mild VI and 22 legally blind patients answered the questionnaire to investigate concurrent validity. The questionnaire was paper-based and was administered through face-to-face interviews. A 5-point Likert scale response format was used to rate the difficulty as follows: (1) no difficulty; (2) little difficulty; (3) some difficulty; (4) great difficulty; and (5) unable to do because of my vision. The same response format was used to evaluate the need for help to perform the activities because of the visual problem: (1) never; (2) rarely; (3) sometimes; (4) often; (5) always. The driving item was rated as follows: (1) yes; (2) no, because of my vision; (3) no, because of other reasons unrelated to my vision.

Responses for each item are converted to a score between 0 and 100 with high scores representing better vision-related functioning. The score of the LIFE4LVQ items was calculated for further analysis and comparison among the three groups. The overall and subscale scores were computed by averaging all relevant scored items and rescaling to a range of 0 to 100 where 0 = unable to do because of my vision/always, and 100 = having no difficulty/never. Items that were ‘‘not applicable’’ were coded as zero so as not to be included in the analysis (note: the number of ‘‘not applicable’’ responses was small).

Furthermore, participants responded to two items with a rating scale from 0 to 10 (0 representing very poor, and 10 excellent) related to self-reported overall health and general vision. The time of completion of the questionnaire was calculated at around twenty minutes.

### 2.3. Rasch Analysis

Rasch analysis [48,49] was performed to assess the psychometric properties of the LIFE4LVQ, which is considered the gold standard method when developing and validating a questionnaire [26,27,50]. The Andrich Rating Scale Model (joint maximum likelihood estimation) was employed using Winsteps software 5.1.7 [25,51]. The examined parameters included: (1) response category functioning, by using category probability curves to test whether the five response categories were adequately ordered [51]. In case of disordering in the “average measure” values, and of mean square fit statistics much larger than 1.0 (indicating misfitting), the substantive disordering of the categories is flagged [51]; (2) item fit statistics (infit and outfit mean square (MNSQ)) were used to confirm the degree to which the data fit the model’s expectations [52]. The scientific literature suggests that fit statistics values must range between 0.70 and 1.30 logits (logarithm of the odds ratio) [12,53]; (3) measurement of precision in terms of person and item reliability (separation index), as indices of the ability of the questionnaire to discern persons along the measured variable [12]. Values greater than 0.8 (>2.0 logits) and 0.9 (>3.0 logits), respectively, are considered acceptable for discrimination of at least 3 strata of persons’ level of the trait being measured [54]; (4) Differential Item Functioning (DIF) which shows whether different subgroups (stratified by age, gender, etc.) have systematically different responses to particular items, despite having equal levels of the trait being measured [55]. The DIF contrast is the difference in the difficulty of the item between the two subgroups and the cutoff of a DIF > 1 logits was used for identifying notable DIF across subgroups of age (above/below median) and gender (male/female) [55]. The Rasch–Welch *t*-test method was used to establish the significance of the DIF contrast; (5) targeting, by examining the item–person map which illustrates differences between a person’s abilities and item difficulty. A difference greater than 1 logit indicates significant mistargeting while a difference of zero characterizes a perfectly-targeted instrument [56]; (6) Principal Component Analysis (PCA) of the residuals was also used to explore the unidimensionality of the measured trait. The LIFE4LVQ was developed to measure two traits: (a) the vision-related ability and (b) the independence of LV patients, as variables of vision-related functionality [23]. The PCA of the residuals was further examined for each construct, separately. The variance explained by the measures for the empiric calculation should be comparable with that of the model (>50%) and the unexplained variance in the first contrast of the residuals less than 2.0 eigenvalue units as an indication of unidimensionality [50].

### 2.4. Statistical Analysis

To assess the convergent validity of the LIFE4LV questionnaire, correlation with BCVA was examined using Spearman’s correlation coefficient [12]. A correlation between 0.3 and 0.9 is considered to be adequate [19]. Discriminant validity illustrates the diversity of two instruments that should be dissimilar [12]. Forty-two LV participants also answered the Greek version of the VFQ-25 questionnaire [57] which captures the QoL rather than activity limitation, to examine the discriminant validity. Concurrent validity refers to the extent to which the instrument can distinguish clinically different groups and it was also evaluated in this study (group 1 mild VI, group 2 LV, and group 3 legally blind participants) [12].

Additionally, thirty LV participants completed the LIFE4LVQ twice with an interval of three weeks to examine test–retest reliability, using Intraclass Correlation Coefficients (ICC). The test–retest reliability reflects the variation in results taken by an instrument on the same subject under the same conditions. Values above 0.75 indicate good to excellent reliability [58]. Statistical analysis was performed using IBM SPSS Statistics for Windows, Version 26.0 (Armonk, NY, USA: IBM Corp). A p-value of <0.05 was considered to indicate statistical significance.

## 3. Results

### 3.1. Participant Characteristics

A total of 294 participants completed the 49-item version of the LIFE4LVQ, of whom 252 were LV patients (50.4% females) with a mean age of 70.1 (SD 16.0) years and with a mean BCVA of 0.70 (SD 0.27) logMAR. The most common cause of LV was Age-related Macular Degeneration (AMD) (43.6%), followed by diabetic retinopathy (24.8%), glaucoma (10.8%), retinitis pigmentosa (5.2%) and other ocular diseases causing irreversible vision loss (Table 2). Sociodemographic characteristics are also shown in Table 2. There was a significant difference in age among the different groups (p = 0.005). The self-reported General Vision (GV) of the LV group was significantly lower than the GV of the normal group (p < 0.001) and significantly higher than the GV of the legally blind group (p = 0.014). Furthermore, there were no significant differences among patients regarding their general health (GH) (p = 0.652).

### 3.2. Unidimensionality

Nine (9) items were found to be misfits (questions: 17, 23, 28, 38, 45, 46, 47, 48, and 49) with infit mean scores of 1.6 and were removed iteratively in the following order: 45, 48, 49, 46, 47, 38, 28, 17, and 23. The removal of these items improved the fit of the scale to the Rasch model. Table 3 summarizes the infit and outfit MNSQ values for each of the 40 remaining items that were included in the LIFE4LVQ. Both the infit and outfit MNSQ values for each of these items fell within the suggested range (0.7–1.3). Item fit statistics for the two subscales of the measurements are shown in Supplementary Materials (Tables S4 and S5).

Furthermore, the PCA of the residuals explained 66.2% (>50%) of the raw variance, but the unexplained variance in 1st contrast was equal to 4.3 eigenvalue units, suggesting a multidimensional scale (the scale measures two constructs: (a) ability and (b) independence). The PCA was further evaluated for each of the two subscales. For the ability subscale, the PCA of the residuals explained 67.3% (>50%) of the raw variance, and the unexplained variance in 1st contrast was 1.9 (<2) eigenvalue units, suggesting unidimensionality. For the independence subscale, the PCA of the residuals explained 67.2% (>50%) of the raw variance. The unexplained variance in 1st contrast was 1.9 (<2) eigenvalue units, also suggesting unidimensionality of the subscale.

### 3.3. Differential Item Functioning (DIF)

The DIF contrast results are also shown in Table 3. DIF was absent (<1 logit) for age and gender which suggests that the items of the measure function equivalently for participants, independently of their age and gender. Furthermore, DIF was also evaluated for each subscale separately and it was absent for all items, both for the ability and independence subscales (Supplementary Tables S4 and S5).

### 3.4. Reliability

The Person Separation Index (PSI) was 5.18 logits (>3.00), and the item reliability value was 0.96 (>0.9), indicating the high measurement precision of the questionnaire. The PSI and reliability were also high for ability (PSI = 4.02 with a reliability of 0.94), and for independence (PSI = 3.51 with a reliability of 0.92), implying the good discriminant ability of both subscales of the measure.

### 3.5. Response Category Functioning

**Figure 1:**
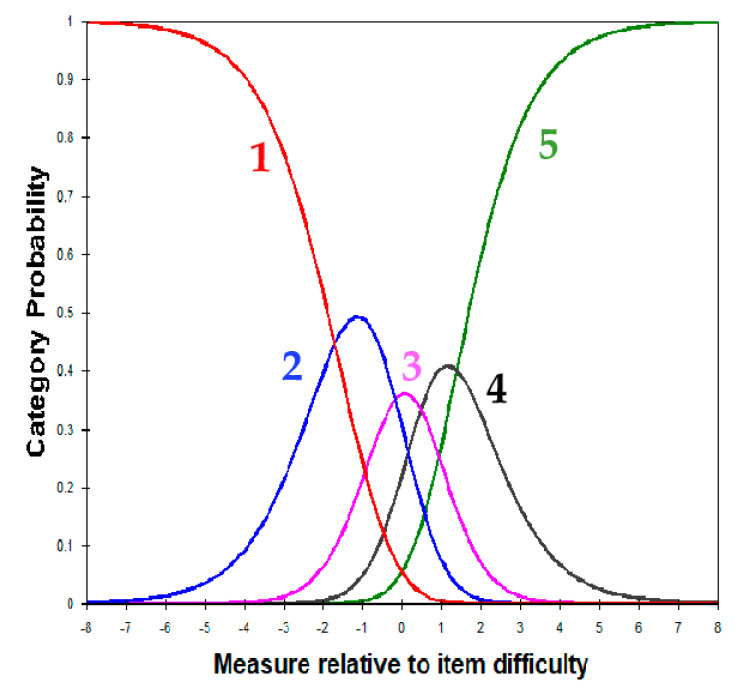
Category probability curves (CPCs) for the LIFE4LVQ. Each curve illustrates one response category (no difficulty/never = 1, little difficulty/little = 2, some difficulty/sometimes = 3, great difficulty/often = 4, and unable to do because of my vision/always = 5). The point where two adjacent curves overlap is the threshold. Thresholds in this case are adequately ordered.

The Category Probability Curves (CPCs) showed a good ordering of category responses (Figure 1). Furthermore, the “Average Measure” values were found to advance with response categories and the category MNSQ fit statistics did not markedly exceed the model values of 1.0, indicating that the original rating scale functioned well (Table 4). The CPCs for each of the measure’s subscales (ability and independence) are shown in Supplementary Figures S1 and S2 and Tables S2 and S3.

Satisfactory category statistics indicate that each category response has an equal probability to be observed. Average measures and thresholds advance with categories and the MNSQs are near 1.0 for each category MNSQ: mean-square fit statistics.

### 3.6. Person–Item Map

**Figure 2:**
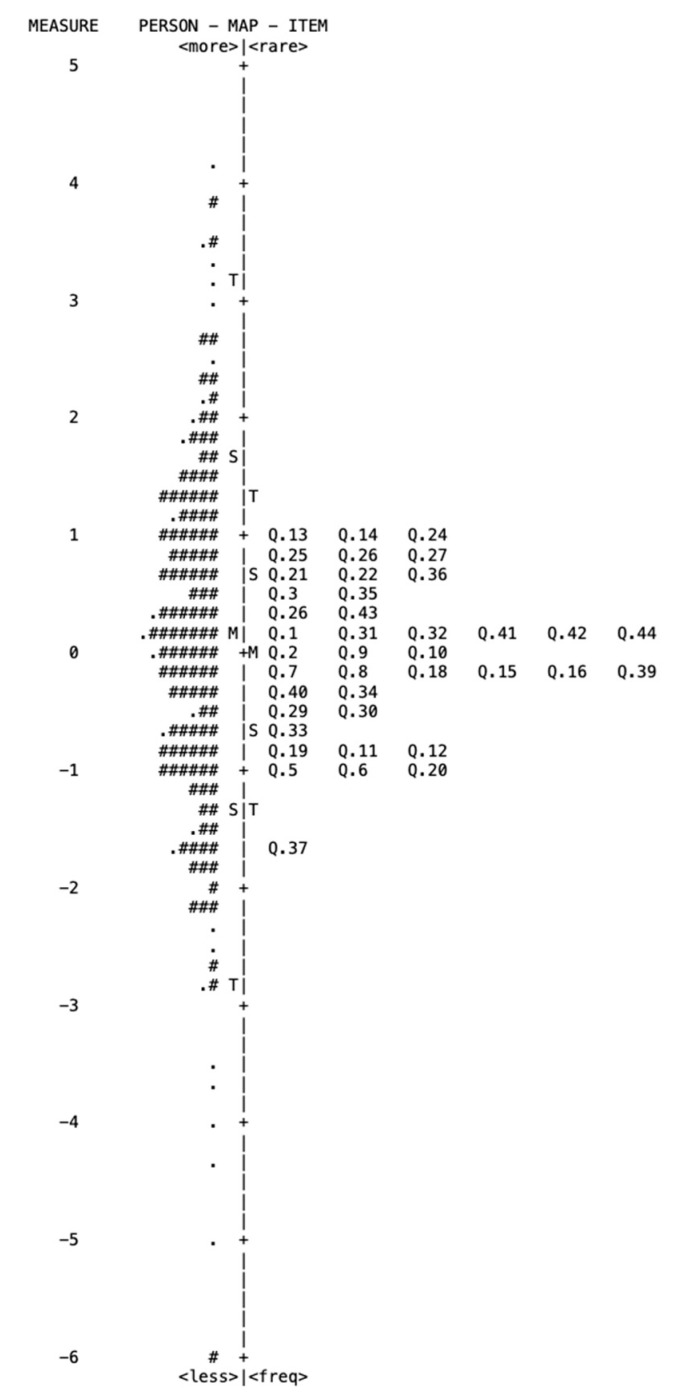
Person–item map for the LIFE4LVQ. Participants (person ability) are presented on the left and the items (item difficulty) are on the right of the dashed line. Each “#” and “.” represent two participants and one participant, respectively. “Q” means the LIFE4LVQ item, and “M” indicates the mean measure (left, person ability; right, item difficulty). “S” shows 1 SD from the mean and “T” indicates 2 SDs, all expressed in logits. SD = standard deviation.

As indicated by the item–person map (Figure 2), the difference between item difficulty and person ability was within the acceptable limits (0.26 logits). This means that the instrument showed good targeting of the 40 items to the responders. The person–item map for both the ability and the independence subscales also revealed that the distribution of item difficulties closely matched the distribution of person abilities (Supplementary Figures S3 and S4).

### 3.7. Validity

The LIFE4LVQ exhibited excellent convergent validity since there was a significant correlation (rho = 0.559, p < 0.001) between logMAR BCVA and the scores obtained in the questionnaire. As the scores of the questionnaire increased, the BCVA also increased (Supplementary Table S6). Regarding the discriminant validity, no significant association was found between the LIFE4LVQ and the NEI-VFQ-25 (Pearson r = −0.103, p = 0.441).

As for concurrent validity, the mean LIFE4LVQ score for subjects with LV was 48.13 ± 28.27 versus 92.43 ± 20.36 for the group with mild VI. Legally blind subjects obtained very poor scores, with a mean of 7.22 ± 5.30. These results indicate statistically significant differences (p < 0.001) between the groups (LV subjects versus subjects with mild impairment, and LV subjects versus legally blind subjects) (Supplementary Table S7).

As for test–retest reliability, the ICC was 0.96 (p < 0.001; 95% CI, 0.93–0.98), indicating high repeatability of the questionnaire (Supplementary Table S8).

## 4. Discussion

The aim of this study was to develop and assess the psychometric properties of the LIFE4LVQ, a new instrument that measures the residual ability and independence as variables of functionality in LV patients. The Rasch-based approach seems to facilitate item-level interpretation, which allows more precise identification of the impact of LV on patients’ ability and independence [26,27,50]. The questionnaire assessed all participants similarly, independently of age and gender. Both subscales of the instrument, ability and independence, demonstrated unidimensionality, which confirms that the questionnaire examines only two constructs. The instrument has good convergent validity; poorer BCVA was significantly associated with worse scores on the LIFE4LVQ [12]. Concerning concurrent validity, patients with LV obtained significantly (p < 0.001) lower scores than participants with mild VI and significantly higher (p < 0.001) than those with legal blindness. Finally, the questionnaire was shown to be highly repeatable, which is a fundamental property for evaluating changes in functionality, when considering a specific intervention [58]. In general, the LIFE4LVQ adequately fits the Rasch model, and it can be used as a valid and repeatable measure, which can accurately detect restrictions on the ability and independence of LV patients due to various causes of irreversible vision loss.

AMD was found to be the main cause of LV in this study, followed by diabetic retinopathy and glaucoma. This finding is consistent with previous studies, suggesting the incidence of ocular diseases causing LV in industrialized countries [3,59]. Moreover, in the present study, most of the LV participants (n = 140, 60.4%) had only a primary education level. There is a well-established link between low literacy level and VI [60].

In the area of rehabilitation, evidence suggests that functionality is mostly improved by the provision of low-vision services [33,36,61]. Various questionnaires have been developed in ophthalmology with different objectives. Most questionnaires target patients with VI in general or with cataract [16] and measure QoL rather than functionality. Massof et al. proposed 377 activities, relevant to LV patients that could be included in a functional questionnaire [23]; however, a tailor-made questionnaire requires a lot of time to be completed and cannot be implemented in clinical settings. Khadka and colleagues recommended the use of the Veterans Affairs Low Vision Visual Function Questionnaire (VA LV VFQ) for activity limitation assessment in adults with LV, as superior among the six instruments with interval scaling in LV [19]. Although the instrument includes the main activities of daily living, it does not capture the independence of LV patients as a significant variable of functionality, and items were not selected according to their significance in LV patients’ lives. Moreover, the USA veteran cohort may not represent the wider visually impaired population. Specifically, the sample consisted mainly of males (270/367 subjects) and the range of visual acuity (VA) of the study group was wider, also including participants with VA near to normal or with legal blindness [62].

The objective of the NEI VFQ-25 was to measure vision-specific QoL in clinical research [33,34,35]. In our study, no significant association was found between the LIFE4LVQ and the VFQ-25 (Pearson r = −0.103, p = 0.441). LIFE4LVQ measures vision-related activity limitation (ability) and the need for help during these activities (independence). The NEI-VFQ-25 captures dimensions of vision-specific QoL, also including the impact of general health, ophthalmic irritations, and driving, an item that was eliminated in our questionnaire based on Rasch analysis. The target group of the LIFE4LVQ is LV patients who are not eligible to drive in many countries, and this item would produce considerable missing data [63]. Previous research supports that the psychometric properties of NEI VFQ-25, as a measure of vision-related QoL, can be improved [16,63,64]. Petrillo and colleagues re-examined the psychometric properties of the NEI VFQ-25 using Rasch analysis, and demonstrated that the optimal structure was 28 items in two subscales: activity limitation (19 items) and socio-emotional functioning (9 items) [64]. A short version of the NEI-VFQ consisting of 7 items demonstrated responsiveness to LV functionality; however, dimensionality was not evaluated during the study [19]. Furthermore, the questionnaire was developed without taking input from LV patients [19]. Rasch analysis revealed problems with the unidimensionality of the VFQ-25, but also with the psychometric properties of VF-14, LVQOL, and MacDQoL—all of them widely used vision-related QoL questionnaires [15,64,65,66]. The MacDQoL, a questionnaire specifically targeting patients with maculopathies, was found to be multidimensional and has a complicated response format [65]. The VF-14 was developed to assess functional limitations caused by cataract and the outcomes of cataract surgery [38,39].

Although some items from these questionnaires could be applied to LV patients, the content or the number of such items in each instrument seem insufficient to capture the range of activities that are affected by LV [31]. Furthermore, the already existing questionnaires lack items related to new technologies, such as the use of digital devices or digital reading. Moreover, there is also a lack of items regarding the activities at night/in low luminance conditions (e.g., mobility), or items that capture the level of independence during the performance of vision-related activities. The LIFE4LVQ was developed as an LV-targeted functionality measure that could be used to tailor rehabilitation programs according to patient needs and to quantify outcomes of rehabilitation programs or other interventions (anti-vascular endothelial growth factor injections, etc.).

Our study has several strengths. To our knowledge, this is the first questionnaire for patients with LV (according to the WHO definition of LV), to investigate functionality in terms of ability and independence, also utilizing Rasch analysis from its conception [19]. Modern psychometric methods such as Rasch analysis provide a more robust approach for the evaluation of validity and interpretability compared to classical psychometric methods [19], eliminating some defects of the traditional methods. Furthermore, the commonly used, vision-related questionnaires almost exclusively focus on items related to functional vision under daytime conditions. In the LIFE4LVQ there are several items related to activities such as mobility at night and under low environmental light levels. Self-reported, low luminance visibility problems are well defined, even in individuals who report relatively good vision-related functioning during daytime conditions [32]. Additionally, to our knowledge, this is the first questionnaire to capture to great extent the variable of independence during the performance of most vision-related activities. Low vision has a detrimental impact on affected individuals’ independence to perform basic self-care activities, such as eating and dressing, as well as instrumental activities of daily living, such as shopping [31]. Last but not least, the process of item selection ensures that items that are irrelevant to the visual impairment were not included in the questionnaire, and special attention was paid to the significance of these activities in patients’ lives [23].

Several limitations must also be acknowledged. There are potential administration and sampling biases. The non-probability sampling can be time-saving and persons who choose to participate are likely to be committed to the research and likely to provide more truthful responses. Although the non-probability sample (applying eligibility criteria) is a widely used method in hospitals or outpatient clinics, this type of sampling is linked to selection bias which can lead to a non-representative sample [67]. Moreover, faking bad performance and falling good performance, respectively, would be potential administration biases. The completion of the scale through an interview is a method to avoid that kind of bias [67]. Our study was a single-center survey, similar to other related studies [16,19]. In our study, each patient who was referred to our unit and fulfilled the eligibility criteria was informed and could participate in the study. Furthermore, another limitation is that the effect of systemic comorbidities, such as arthritis, cardiovascular disease, hypertension, diabetes, chronic renal disease, cancer, stroke, etc., was not evaluated. However, there were no significant differences among patients of different groups regarding their general health (GH) (p = 0.652), suggesting that the overall score reflects only the impact of LV. Future work is required to validate the questionnaire in other languages to be used in other populations.

## 5. Conclusions

In summary, the 40-item LIFE4LVQ was developed using Rasch-based approaches and is a psychometrically valid measure to evaluate the impact of LV on the functionality of affected individuals. The LIFE4LVQ may be useful to clinicians who want to quantify the self-reported functional status of LV patients to improve the offered services. The LIFE4LVQ may provide significant information to researchers who intend to design and evaluate rehabilitation strategies based on PROMs feedback. Future work is needed to validate the LIFE4LV questionnaire in other languages.

## Figures and Tables

**Table 1:** Self-reported daily activities of LV patients (N = 110) *.

Daily Activities	Ν (%)
Technology-Communication	32 (29.1)
Cooking	43 (39.1)
Watching Television (TV)	27 (24.5)
Self-care	27 (24.5)
Social functioning	38 (34.5)
Housekeeping	39 (35.5)
Reading ^‡^	47 (42.3)
Books/Hardcopies	32 (68.1)
Digital Texts	29 (61.7)
Labels	22 (46.8)
Prices	19 (40.4)
Bills	12 (25.5)
Subtitles	11 (23.4)
Going out/Walking	39 (35.5)
Public Transport use	24 (21.8)
Sex life	2 (1.8)
Travelling	12 (10.1)
Spirituality-Praying	3 (2.7)
Shopping	22 (20)
Family	12 (10.1)
Gardening	12 (10.1)
Work/Education	28 (25.5)
Pets	4 (3.6)
Needlecraft	4 (3.6)
Driving	6 (5.5)
Watching Theater	2 (1.8)
Volunteering	2 (1.8)
Flirting	12 (10.1)

Responses of LV participants participating in phase 1 and used to identify content for item generation. * Each participant reported up to three significant activities affected by LV. ^‡^ Reading was further analyzed into subsections according to the statements of 47 participants who reported reading as a significant activity affected by LV. N: number of participants.

**Table 2:** Clinical and sociodemographic characteristics of the participants.

	Low Vision N = 252 (%)	Legally Blind N = 22 (%)	Mild Visual Impairment N = 20 (%)	p *
Age group, N (%)				0.005
<55	35 (13.5)	8 (36.4)	1 (5)	
55–74	92 (38.5)	6 (27.2)	9 (45)	
≥75	125 (48)	8 (36.4)	10 (50)	
Gender, females, N (%)	129 (50.4)	10 (45.5)	11 (61.1)	0.676
Educational level, Ν (%)				0.816
Primary	150 (59.5)	12 (54.5)	7 (46.7)	
Lower and upper secondary	53 (21)	7 (31.8)	11 (40)	
Higher	49 (19.4)	3 (13.6)	2 (13.3)	
General health (mean (SD), range)	6.31 (2.16), 0–10	5.86 (2.76), 1–10	6.30 (1.92), 2–9	0.652
General vision (mean (SD), range)	4.88 (1.89), 0–9	2.68 (2.23), 0–8	6.10 (1.62), 2–8	<0.001
BCVA of the better-seeing eye (logMAR, mean (SD), range)	0.70 (0.27), 0.5–1.3	1.53 (0.09), 1.3–1.6	0.16 (0.13), 0.0–0.4	
Ocular disease, Ν (%)				
Age-related Macular Degeneration (AMD)	106 (42)			
Diabetic Retinopathy (DR)	62 (24.6)			
Glaucoma	29 (11.5)			
Retinitis Pigmentosa (RP)	13 (5.2)			
Other	42 (16.6)			

* p-values < 0.05 indicate statistically significant differences. Other: retinal detachment-treated (n = 9), optic nerve atrophy (n = 9), epiretinal membrane (n = 8), Leber congenital amaurosis (n = 3), retinal vein occlusion (n = 3), central vein occlusion (n = 2), Stargardt disease (n = 3), macular hole (n = 1), choroideremia (n = 1), Fuchs’ dystrophy (n = 2), and birdshot chorioretinopathy (n = 1). N: number of participants, SD: Standard Deviation, BCVA: Best Corrected Visual Acuity, logMAR: logarithm of the Minimum Angle of Resolution.

**Table 3:** Estimates of item measures and fit statistics from Rasch analysis for the LIFE4LVQ.

Items	Measure	SE	Infit MNSQ	Outfit MNSQ	Age DIF Contrast ^a^ *	Gender DIF Contrast ^a^
Shopping (A)	0.21	0.07	0.85	0.96	−0.05	0.08
Shopping (I)	0.03	0.07	1.01	0.99	0.00	0.14
Product recognition (A)	0.51	0.07	0.81	0.92	−0.31	−0.22
Product recognition (I)	0.38	0.07	1.04	0.97	−0.32	−0.24
Price identification (A)	−0.91	0.08	0.92	0.92	0.21	0.02
Price identification (I)	−0.96	0.08	1.07	0.92	0.17	0.09
Use of electronic devices (A)	−0.21	0.08	1.07	1.07	0.35	−0.24
Use of electronic devices (I)	−0.14	0.08	1.28	1.19	0.37	−0.20
Use of public transport—daytime (A)	0.09	0.08	0.81	0.78	0.04	0.24
Use of public transport—daytime (I)	0.09	0.08	1.02	0.89	0.11	0.34
Use of public transport—night (A)	−0.84	0.08	0.87	0.83	0.20	0.42
Use of public transport—night (I)	−0.77	0.08	1.24	1.08	0.22	0.36
Walking in familiar places—daytime (A)	1.02	0.08	0.73	0.73	0.15	0.20
Walking in familiar places—daytime (I)	0.98	0.08	1.06	0.99	0.02	0.27
Walking in familiar places—night (A)	−0.2	0.07	1.03	1.02	−0.06	0.23
Walking in familiar places—night (I)	−0.18	0.07	1.3	1.17	0.00	0.47
Walking in unfamiliar places—daytime (I)	−0.15	0.07	0.87	0.77	0.00	0.19
Walking in unfamiliar places—night (A)	−0.9	0.08	0.99	1.03	−0.24	0.23
Walking in unfamiliar places—night (I)	−0.98	0.08	1.24	1.17	−0.10	0.44
Noticing objects around (A)	0.72	0.07	1.1	1.17	−0.49	−0.11
Noticing objects around (I)	0.73	0.07	1.17	1.06	−0.60	−0.10
Preparing meals and drinks (I)	0.96	0.08	0.99	0.86	−0.06	−0.06
Matching clothes according to color (A)	0.9	0.08	0.91	0.95	0.00	−0.41
Matching clothes according to color (I)	0.88	0.08	1.21	1.12	0.05	−0.65
Self-care (A)	0.79	0.07	1.22	1.3	−0.29	−0.43
Reading relatively large letters in formal texts (A)	−0.42	0.07	1.09	0.97	0.38	0.08
Reading relatively large letters in formal texts (I)	−0.48	0.07	1.3	1.13	0.41	0.00
Working/Studying (A)	0.1	0.08	0.86	0.91	−0.22	−0.28
Working/Studying (I)	0.09	0.08	0.92	1.08	−0.24	−0.16
Reading relatively large letters in magazines etc. (A)	−0.58	0.08	1.11	1.08	0.56	0.06
Reading relatively large letters in magazines etc. (I)	−0.36	0.08	1.22	1.02	0.44	0.13
Watching television (A)	0.46	0.07	1.04	1.06	0.15	−0.11
Watching television (I)	0.71	0.08	1.03	0.97	0.15	0.00
Reading subtitles (A)	−1.72	0.09	1.13	0.92	0.00	−0.02
Traveling (A)	−0.14	0.08	0.74	0.75	0.26	−0.18
Traveling (I)	−0.3	0.08	1.01	0.9	0.08	0.06
Recognizing faces/reactions (A)	0.08	0.07	0.91	0.94	−0.08	−0.38
Recognizing faces/reactions (I)	0.12	0.07	0.94	1.0	−0.20	−0.33
Social life (A)	0.25	0.07	0.75	0.7	−0.25	0.00
Social life (I)	0.14	0.07	1.03	0.89	−0.45	0.18

S.E. = Standard Error, MNSQ: Mean Square, DIF: Differential Item Functioning. (A) = ability to perform the task/activity with residual vision; (I) = need help to perform the task/activity with residual vision. ^a^ Welch’s test, DIF contrast: difference in DIF size between the two subgroups in logits. * Age group (above/below the median = 72 years).

**Table 4:** LIFE4LVQ categories and Andrich thresholds.

LIFE4LVQ Response Categories	Count	Average Measure	Infit MNSQ	Outfit MNSQ	Andrich Threshold
1	2112	−1.73	1.20	1.19	None
2	1963	−0.80	0.88	0.87	−1.18
3	1570	0.04	0.88	0.78	−0.11
4	1866	0.85	0.93	0.90	0.26
5	2418	1.81	1.11	1.12	1.03

## Data Availability

Data could be made available through communication with the corresponding author.

## Supplementary Materials

The following supporting information can be downloaded at: https://www.mdpi.com/article/10.3390/jcm12072549/s1, Figure S1: Category Probability Curves (CPCs) for the Ability subscale; Figure S2: Category Probability Curves (CPCs) for the Independence subscale; Figure S3: Person-Item Map for the Ability Subscale; Figure S4: Person-Item Map for the Independence Subscale Table S1: Item Content of the LIFE4LV Questionnaire; Table S2: Ability Categories and Andrich Thresholds; Table S3: Independence Categories and Andrich Thresholds; Table S4: Estimates of item measures and fit statistics from Rasch analysis for the Ability subscale; Table S5: Estimates of item measures and fit statistics from Rasch analysis for the Independence subscale; Table S6: Spearman’s correlations between the overall and subscales score and BCVA (logMAR); Table S7: Overall and subscales scores for the different groups; Table S8: Test-Retest Reliability.
