# Supplementary material for: Development and Validation of the Life for Low Vision Questionnaire (LIFE4LVQ) Using Rasch Analysis: A Questionnaire Evaluating Ability and Independence
Source: J Clin Med. 2023 Mar 28;12(7):2549. doi: 10.3390/jcm12072549 (PMC10095134; doi:10.3390/jcm12072549)
Supplement: Supplementary file 1 [file jcm-12-02549-s001.zip › jcm-2180400-supplementary.pdf]

Supplementary Material

Table 1. Item Content of the LIFE4LV Questionnaire*

Item No.	Item Description
**1**	Can you go shopping?
**2**	Do you need help for shopping due to your visual impairment?
**3**	Can you recognize the products?
**4**	Do you need help for product recognition due to your visual impairment?
**5**	Can you see the prices?
**6**	Do you need help for price recognition due to your visual impairment?
**7**	Can you use electronic devices such as PC, laptop, smartphones etc?
**8**	Do you need help when you use electronic devices such as PC, laptop, smartphones etc. due to your visual impairment?
**9**	Can you use means of public transport during daytime?
**10**	Do you need help in order to use means of public transport during daytime due to your visual impairment?
**11**	Can you use means of public transport at night or under low luminance conditions?
**12**	Do you need help in order to use public transport at night or under low luminance conditions due to your visual impairment?
**13**	Can you walk in familiar places during daytime?
**14**	Do you need help in order to walk in familiar places during daytime due to your visual impairment?
**15**	Can you walk in familiar places at night or under low luminance conditions?
**16**	Do you need help in order to walk in familiar places at night or under low luminance conditions due to your visual impairment?
**17**	Can you walk in unfamiliar places during daytime?
**18**	Do you need help in order to walk in unfamiliar places during daytime due to your visual impairment?
**19**	Can you walk in unfamiliar places at night or under low luminance conditions?
**20**	Do you need help in order to walk in unfamiliar places at night or under low luminance conditions due to your visual impairment?
**21**	Can you notice objects around you as you walk?
**22**	Do you need help in order to notice objects around you as you walk along due to your visual impairment?
**23**	Can you prepare your meals and drinks?
**24**	Do you feel you need help in order to prepare your meals and drinks due to your visual impairment?
**25**	Can you match your clothes according to their color?
**26**	Do you need help in order to match your clothes according to their color due to your visual impairment?
**27**	Can you take care of yourself (shaving, styling your hair, or putting on makeup etc.)?
**28**	Do you need help in order to take care of yourself (shaving, styling your hair, or putting on makeup etc.) due to your visual impairment?
**29**	Can you read texts with relatively large letters on bills, medicals, or on legal forms etc. with your reading glasses?
**30**	Do you need help in order to read texts with relatively large letters on bills, a medicine bottle, or on legal forms etc. with your reading glasses due to your visual impairment?

**31**	Can you meet your work's/ studies' requirements?
**32**	Do you need help in order to meet your work's/ studies' requirements due to your visual impairment?
**33**	Can you read newspapers/magazines/ texts on the screens with relatively large letters with your reading glasses?
**34**	Do you need help in order to read newspapers/magazines/ texts on the screen with relatively large letters with your reading glasses due to your visual impairment?
**35**	Can you watch your favorite Greek series/ movies/ TV shows from a relatively near distance?
**36**	Do you need help in order to watch your favorite Greek series/ movies/ tv shows from a relatively near distance due to your visual impairment?
**37**	Can you read the subtitles in foreign movies from a relatively near distance?
**38**	Do you need help with the subtitles in foreign movies from a relatively near distance due to your visual impairment?
**39**	Can you travel/go for excursions?
**40**	Do you need help in order to travel/go for excursions due to your visual impairment?
**41**	Can you recognize faces and notice their reactions?
**42**	Do you need help with face and reactions recognition due to your visual impairment?
**43**	Can you participate in social life activities such as visiting restaurants, cafes, parties, concerts, theater etc.?
**44**	Do you need help in order to participate in social life activities such as visiting restaurants, cafes parties, concerts, theater etc. due to your visual impairment?
**45**	Can you drive?
**46**	Can you participate in sport activities (walking, jogging, swimming etc.)?
**47**	Do you need help in order to participate in sport activities (walking, jogging, swimming etc.)?
**48**	Can you flirt?
**49**	Do you need help in order to flirt?

Response Categories of items of ability subscale (items1,3,5,7,9,11,13,15,17,19,21,23,25,27,29,31,33,35,37,39,41,43,45,46,48): 1. without any difficulty; 2. with a little difficulty; 3. with a moderate difficulty; 4. with extreme difficulty; 5. I am not able to do this anymore due to my vision problem, of independence subscale (items2,4,6,8,10,12,14,16,18,20,22,24,26,28,30,32,34,36,38,40,42,44,47,49): 1.never; 2. rarely; 3. sometimes; 4. often; 5. always.
*Translated by an English native speaker from Greek to English for the publication. Formatted questionnaire available in Greek language (supplementary material).

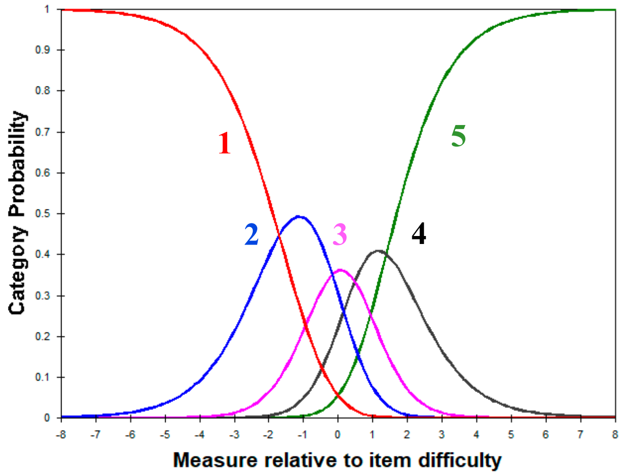

Figure 1. Category probability curves (CPCs) for the Ability subscale. Each curve illustrates one response category (no difficulty =1, little difficult =2, some difficulty =3, great difficulty =4 and unable to do because of my vision=5). The point where two adjacent curves overlap is the threshold. Thresholds in this case are adequately ordered.

Table 2. Ability Categories and Andrich Thresholds

Independence Response Categories	Count	Average Measure	Infit MNSQ	Outfit MNSQ	Andrich Threshold
1	855	-2.04	1.17	1.12	None
2	1100	-0.89	0.92	0.92	-1.70
3	922	0.09	0.88	0.80	-0.17
4	974	1.02	0.89	0.83	0.48
5	1105	2.10	1.11	1.11	1.39

Satisfactory category statistics for the Ability subscale which indicate that each category response has equal probability to be observed. Average measures and thresholds advance with categories and MNSQs are near 1.0 for each category MNSQ: mean-square fit statistics

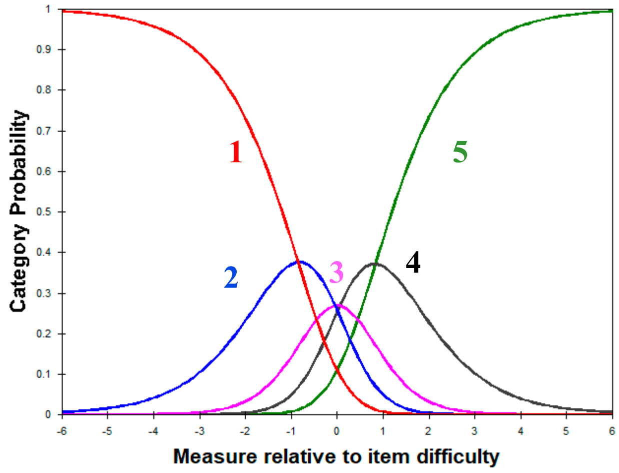

Figure 2. Category probability curves (CPCs) for the Independence subscale. Each curve illustrates one response category (never=1, rarely=2, sometimes=3, often=4 and always=5). The point where two adjacent curves overlap is the threshold. Thresholds in this case are adequately ordered.

Table 3. Independence Categories and Andrich Thresholds

Ability Response Categories	Count	Average Measure	Infit MNSQ	Outfit MNSQ	Andrich Threshold
1	1257	-1.67	1.21	1.25	None
2	863	-0.74	0.87	0.86	-0.85
3	648	0.01	0.86	0.81	-0.04
4	892	0.75	1.00	0.98	0.06
5	1313	1.75	1.08	1.09	0.83

Satisfactory category statistics for the Ability subscale which indicate that each category response has equal probability to be observed. Average measures and thresholds advance with categories and MNSQs are near 1.0 for each category MNSQ: mean-square fit statistics

Table 4. Estimates of item measures and fit statistics from Rasch analysis for the Ability subscale

Items	Measure	SE	Infit MNSQ	Outfit MNSQ	Age DIF Contrast[a*]	Gender DIF Contrast[a]
**Shopping (A)**	0.21	0.07	0.85	0.96	-0.06	0.12
**Product Recognition (A)**	0.51	0.07	0.81	0.92	-0.35	-0.18
**Price Identification (A)**	-0.91	0.08	0.92	0.92	0.24	0.05
**Use of Electronic Devices (A)**	-0.21	0.08	1.07	1.07	0.39	-0.24
**Using Public Transport-daytime (A)**	0.09	0.08	0.81	0.78	0.05	0.34
**Using Public Transport-night (A)**	-0.84	0.08	0.87	0.83	0.23	0.51
**Walk in familiar places-daytime (A)**	1.02	0.08	0.73	0.73	0.17	0.29
**Walk in familiar places-night (A)**	-0.2	0.07	1.03	1.02	-0.08	0.33
**Walk in unfamiliar places-night (A)**	-0.9	0.08	0.99	1.03	-0.28	0.30
**Notice objects around (A)**	0.72	0.07	1.1	1.17	-0.57	-0.06
**Match clothes according to color (A)**	0.9	0.08	0.91	0.95	0.00	-0.40
**Selfcare (A)**	0.79	0.07	1.22	1.3	-0.35	-0.42
**Read relatively large letters on formal texts (A)**	-0.42	0.07	1.09	0.97	0.41	0.14
**Work/Studies (A)**	0.1	0.08	0.86	0.91	-0.24	-0.28
**Read relatively large letters on magazines etc. (A)**	-0.58	0.08	1.11	1.08	0.62	0.11
**Watching Television (A)**	0.46	0.07	1.04	1.06	0.15	-0.06
**Reading subtitles (A)**	-1.72	0.09	1.13	0.92	0.00	-0.01
**Travelling (A)**	-0.3	0.08	1.01	0.9	0.27	-0.13
**Recognize faces/reactions (A)**	0.08	0.07	0.91	0.94	-0.12	-0.36
**Social life (A)**	0.25	0.07	0.75	0.7	-0.28	0.00

S.E. = Standard Error, MNSQ: Mean Square, DIF: Differential Item Functioning
(A) = ability to perform the task/activity with the residual vision
[a]Welch's test, DIF contrast: difference of DIF size between the two subgroups in logits
*Age group (above/below the median=72 years)

Table 5. Estimates of item measures and fit statistics from Rasch analysis for the Independence subscale

Items	Measure	SE	Infit MNSQ	Outfit MNSQ	Age DIF Contrast[a*]	Gender DIF Contrast[a]
**Shopping (I)**	0.03	0.07	1.01	0.99	0.00	0.07
**Product Recognition (I)**	0.38	0.07	1.04	0.97	-0.31	-0.28
**Price Identification (I)**	-0.96	0.08	1.07	0.92	0.16	0.08
**Use of Electronic Devices (I)**	-0.14	0.08	1.28	1.19	0.36	-0.22
**Using Public Transport-daytime (I)**	0.09	0.08	1.02	0.89	0.11	0.28
**Using Public Transport-night (I)**	-0.77	0.08	1.24	1.08	0.22	0.34
**Walk in familiar places-daytime (I)**	0.98	0.08	1.06	0.99	0.02	0.20
**Walk in familiar places-night (I)**	-0.18	0.07	1.3	1.17	0.00	0.40
**Walk in unfamiliar places-daytime (I)**	-0.15	0.07	0.87	0.77	0.00	0.13
**Walk in unfamiliar places-night (I)**	-0.98	0.08	1.24	1.17	-0.08	0.41
**Notice objects around (I)**	0.73	0.07	1.17	1.06	-0.58	-0.15
**Prepare meals and drinks (I)**	0.96	0.08	0.99	0.86	-0.06	-0.11
**Match clothes according to color (I)**	0.88	0.08	1.21	1.12	0.05	-0.68
**Read relatively large letters on formal texts (I)**	-0.48	0.07	1.3	1.13	0.42	-0.04
**Work/Studies (I)**	0.09	0.08	0.92	1.08	-0.23	-0.18
**Read relatively large letters on magazines etc. (I)**	-0.36	0.08	1.22	1.02	0.44	0.10
**Watching Television (I)**	0.71	0.08	1.03	0.97	0.15	-0.02
**Travelling (I)**	-0.3	0.08	1.01	0.9	0.09	0.03
**Recognize faces/reactions (I)**	0.12	0.07	0.94	1.0	-0.18	-0.38
**Social life (I)**	0.14	0.07	1.03	0.89	-0.43	0.12

S.E. = Standard Error, MNSQ: Mean Square, DIF: Differential Item Functioning
(I) = need for help in order to perform the task/activity with the residual vision
[a]Welch's test, DIF contrast: difference of DIF size between the two subgroups in logits
*Age group (above/below the median=72 years)

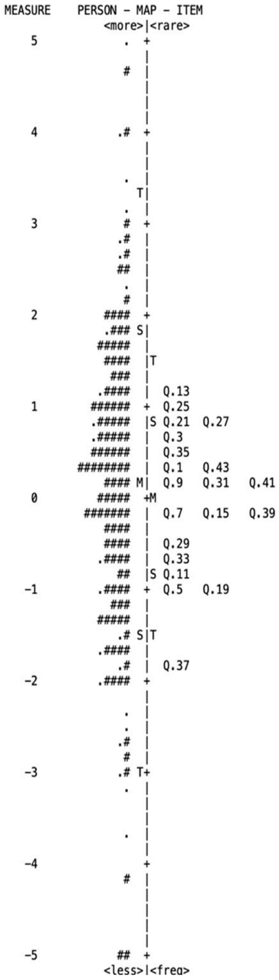

Figure 3. Person-Item Map for the Ability Subscale. Participants (person ability) are presented on the left and the items (item difficulty) on the right of the dashed line. Each "#" and "." represent two and one participants respectively. “Q” means the LIFE4LV item,“M”indicates the mean measure (left, person ability; right, item difficulty). “S”shows 1 SD from the mean and “T” indicates 2 SDs, all expressed in logits

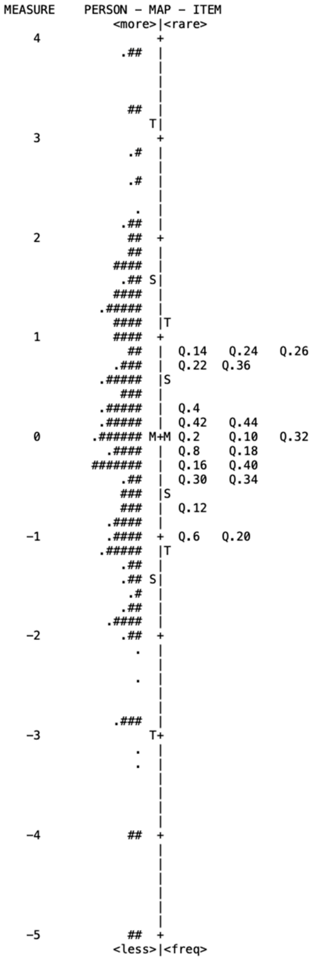

Figure 4. Person-Item Map for the Independence Subscale. Participants (person ability) are presented on the left and the items (item difficulty) on the right of the dashed line. Each "#" and "." represent two and one participants respectively. “Q” means the LIFE4LV item,“M”indicates the mean measure (left, person ability; right, item difficulty). “S”shows 1 SD from the mean and “T” indicates 2 SDs, all expressed in logits

Table 6. Spearman's correlations between the overall and subscales score and BCVA

Subscales*BCVA (LogMAR)	Spearman's rho	p*
**Ability**	-0.559	**<0.001**
**Independence**	-0.554	**<0.001**
**LIFE4LVQ**	-0.555	**<0.001**

*Bolded values indicate statistically significant results (p<0.05).
BCVA: Best corrected Visual Acuity, logMAR: logarithm of the Minimum Angle of Resolution, LIFE4LVQ: Life for (4) Low Vision Questionnaire

Table 7. Overall score and subscale scores for the different groups

Subscale	Number of items	Intraclass correlation coefficient (ICC, 95%CI)
**Ability**	20	0.93 (0.88-0.96)
**Independence**	20	0.97 (0.94- 0.98)
**LIFE4LVQ**	40	0.96 (0.93- 0.98)

ICC: Intraclass Correlation Coefficient, 95% CI: 95% Confidence Intervals, LIFE4LVQ: Life for (4) Low Vision Questionnaire

Table 8. Test-Retest Reliability

	Low Vision		Legally Blind		Mild Visual Impairment		p*
**Subscale**	Mean (SD)	Median (IQR)	Mean (SD)	Median (IQR)	Mean (SD)	Median (IQR)	
**Ability**	47.43 (26.61)	45.0 (26.56, 70.93)	6.45 (6.52)	5.0 (1.12, 10.31)	87.42 (19.63)	92.5 (86.25, 95.62)	**<0.001**
**Independence**	48.47 (30.50)	47.5 (20.62, 76.87)	10.13 (12.09)	6.25 (1.12, 12.5)	91.91 (19.82)	97.5 (92.5, 100.0)	**<0.001**
**LIFE4LVQ**	48.13 (28.27)	46.87 (25.0, 72.5)	7.22 (5.30)	6.87 (2.65, 10.78)	89.67 (19.59)	95.62 (88.75, 97.18)	**<0.001**

*Bolded values indicate statistically significant results (p<0.05).
SD: Standard Deviation, IQR: Interquartile Range, LIFE4LVQ: Life for (4) Low Vision Questionnaire
